# Membrane Environment Enables Ultrafast Isomerization of Amphiphilic Azobenzene

**DOI:** 10.1002/advs.201903241

**Published:** 2020-03-06

**Authors:** Giuseppe Maria Paternò, Elisabetta Colombo, Vito Vurro, Francesco Lodola, Simone Cimò, Valentina Sesti, Egle Molotokaite, Mattia Bramini, Lucia Ganzer, Daniele Fazzi, Cosimo D'Andrea, Fabio Benfenati, Chiara Bertarelli, Guglielmo Lanzani

**Affiliations:** ^1^ Center for Nano Science and Technology Istituto Italiano di Tecnologia Via Pascoli 70/3 20133 Milano Italy; ^2^ Center for Synaptic Neuroscience and Technology Istituto Italiano di Tecnologia Largo Rosanna Benzi 10 16132 Genova Italy; ^3^ IRCCS Ospedale Policlinico San Martino Largo Rosanna Benzi 10 16132 Genova Italy; ^4^ Dipartimento di Fisica Politecnico di Milano Piazza L. da Vinci 32 20133 Milano Italy; ^5^ Dipartimento di Chimica Materiali e Ingegneria Chimica “Giulio Natta” Politecnico di Milano Piazza L. da Vinci 32 20133 Milano Italy; ^6^ Department of Chemistry Institut für Physikalische Chemie University of Cologne Luxemburger Str. 116 D‐50939 Köln Germany; ^7^ Department of Applied Physics Faculty of Sciences University of Granada C/Fuentenueva s/n 18071 Granada Spain

**Keywords:** amphiphilic, azobenzene, cell membranes, cell stimulation, ultrafast isomerization

## Abstract

The non‐covalent affinity of photoresponsive molecules to biotargets represents an attractive tool for achieving effective cell photo‐stimulation. Here, an amphiphilic azobenzene that preferentially dwells within the plasma membrane is studied. In particular, its isomerization dynamics in different media is investigated. It is found that in molecular aggregates formed in water, the isomerization reaction is hindered, while radiative deactivation is favored. However, once protected by a lipid shell, the photochromic molecule reacquires its ultrafast photoisomerization capacity. This behavior is explained considering collective excited states that may form in aggregates, locking the conformational dynamics and redistributing the oscillator strength. By applying the pump probe technique in different media, an isomerization time in the order of 10 ps is identified and the deactivation in the aggregate in water is also characterized. Finally, it is demonstrated that the reversible modulation of membrane potential of HEK293 cells via illumination with visible light can be indeed related to the recovered trans→cis photoreaction in lipid membrane. These data fully account for the recently reported experiments in neurons, showing that the amphiphilic azobenzenes, once partitioned in the cell membrane, are effective light actuators for the modification of the electrical state of the membrane.

## Graphical abstract

The use of light to achieve control of cellular signaling is an important topic in pharmacology. Here, it is shown that an amphiphilic azobenzene performs ultrafast isomerization when partitioned into the cell membrane, while in water its photoisomerization ability is lost because of the formation of excimeric aggregates. This enables fast and reversible modulation of the membrane potential.

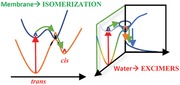

The precise, fast, and efficient spatiotemporal control of cellular signaling via the use of external triggers has become an important topic in chemical biology[qv: 1] and photopharmacology.[qv: 2,3] In this regard, light‐responsive systems offer the possibility to employ light as a clean, non‐invasive, and spatio‐temporally precise tool for controlling a variety of biological signals both in vitro and in vivo.[qv: 4–9] A plethora of photochromic systems allowing modulation of molecular responses in a reversible fashion have been developed.[qv: 10,11] Among them, azobenzenes[qv: 12] are widely employed as versatile photoresponsive element for conferring reversible sensitivity to bio(inspired) targets and drugs, such as artificial membranes,[qv: 13–15] peptides,[qv: 16,17] nucleic acids,[qv: 18,19] ion channels,[qv: 20,21] living organisms,[qv: 22] and antibacterial molecules.[qv: 23–25] These studies have demonstrated that effective and reversible photomodulation can be achieved both in vitro and in vivo, by the covalent binding of the azobenzene unit to the selected target. Alternatively, one can exploit non‐covalent interactions to assist the incorporation of photoactive molecules in bio‐systems.[qv: 26–30] Although this method is less specific than the covalent approach, the relatively weak non‐covalent interactions still allow for driving affinity to various bio‐interfaces, while ensuring full reversibility and much lower invasiveness. For instance, the ability of amphiphilic azobenzenes to intercalate spontaneously in model artificial membranes and micelles has been used to guide their internalization into these systems, mostly to study intrinsic membrane properties,[qv: 15,31] to build up photoresponsive cargos for drug delivery applications,[qv: 32,33] or to provide novel antibacterial systems.[qv: 25] Very recently a new amphiphilic azobenzene molecule named ZIAPIN2 has been introduced. ZIAPIN2 predominantly localizes in the plasma membrane and potently modulates neuronal firing in vitro as well in vivo via an opto‐mechanical effect.[qv: 34] In order to fully characterize the photostimulation mechanism of ZIAPIN2, here we report a detailed investigation of its photophysics and dynamics in different media. By combining steady‐state and time‐resolved optical spectroscopies, we observed that ZIAPIN2 in water lends itself to a radiative transition likely mediated by the formation of excimers, whereas it reacquires its conformational dynamics in membrane‐mimicking environments. Characteristic time scales and reaction yields are reported, reconciling the fast response (ms) observed in functional studies and the apparent slow ensemble evolution(s) by determining the molecular conversion time in the tens of picosecond range. Finally, we validate this opto‐mechanical phenomenon in a non‐excitable cell line that has an intrinsically low concentration of ion channels, to single out the effect on the plasma membrane itself.

ZIAPIN2 was synthesized by reduction of the commercially available Disperse Orange 3 followed by the substitution of the amines with an excess of 1,6‐dibromohexane. Full details about the synthesis can be found in ref. 34 and Supporting Information. The molecular structures and electronic transition energies of ZIAPIN2 were computed at the density functional theory (DFT) and time‐dependent density functional theory (TDDFT) level (see Supporting Information), for both trans and cis isomers (**Figure** **1**a). The trans species shows a quasi‐planar structure with a dihedral angle between the phenyl rings of 14°; the cis shows a distorted structure, with a dihedral angle of 46°. The computed vertical transition energies well agree with the experimental UV–vis absorption spectra reported in Figure 1b. In vacuum, the trans species shows a vertical (i.e., Franck–Condon region) dipole active excited state at 430 nm (*f* = 1.33) featuring an n–π* character with molecular orbitals delocalized over the azobenzene moiety. The cis species shows two vertical dipole active excited states computed at 537 nm (*f* = 0.18) and 367 nm (*f* = 0.33), associated with the n–π* and π–π* transitions, respectively. Note that the diamino substitution induces a red shift of the optical gap with respect to the unsubstituted azobenzene that is favorable to biological applications. As discussed in the following sections, the addition of water induces a broadening and a red‐shift of the absorption spectrum of the trans species. To a first approximation, we performed DFT/conductor‐like polarizable continuum model calculations on the single molecule. Considering water as a solvent, two active states are computed at 456 nm (*f* = 1.34) and 436 nm (*f* = 0.15), the former being red‐shifted by 26 nm with respect to the active state in the vacuum case. We preliminary speculate that such a shift in the ground state absorption might be related to the solvent cage (in this case water) that can play a role in the molecular conformation. As shown later, however, modeling at the single molecule level is not sufficient to fully account for the observed phenomena.

**Figure 1:**
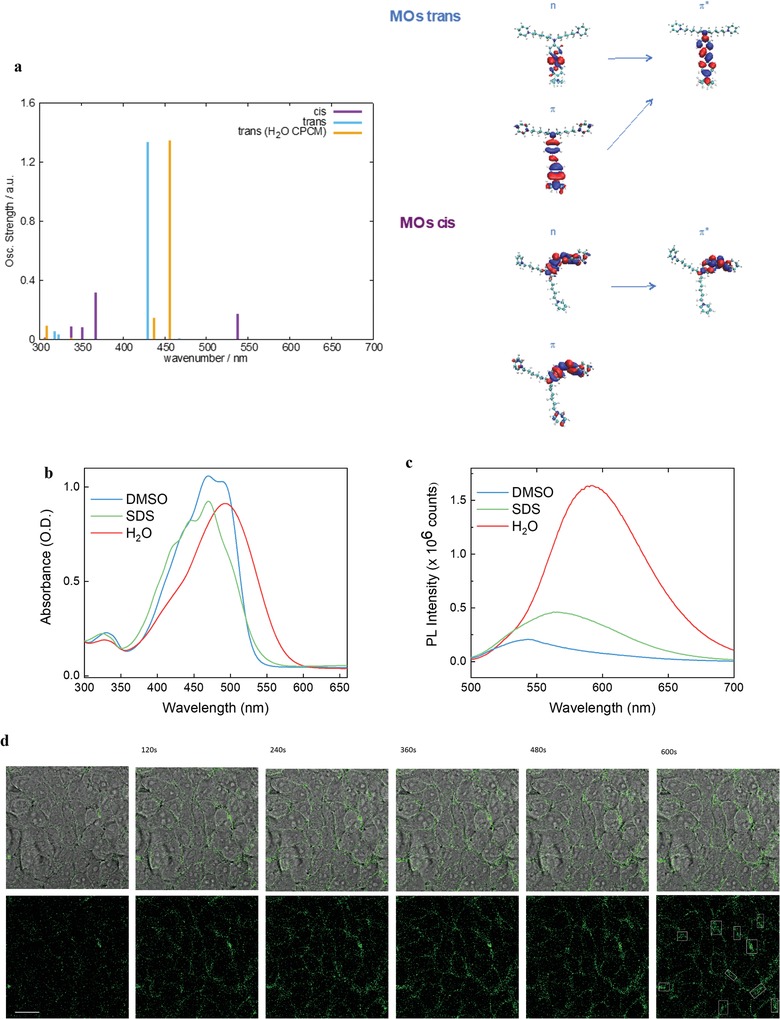
a) Computed UV–vis electronic transition and molecular structures for trans and cis ZIAPIN2 at the DFT and TDDFT level of theory (see Supporting Information for details). b) UV–vIS and c) PL spectra of ZIAPIN2 in DMSO, SDS 100 mm, and water (25 µm). PL spectra were normalized to both lamp intensity and ground state absorption, to obtain a relative PL quantum yield among the three solutions. d) Time‐lapse bright‐field and fluorescence confocal images of ZIAPIN2 (in DMSO 25 µm) loading in HEK293 cells (scale bar = 50 µm).

We used UV–vis absorption and photoluminescence (PL) spectroscopies to investigate the behavior of ZIAPIN2 in the following media: i) DMSO that is a good solvent, as a control medium and vector for ZIAPIN2; ii) water that is the main component of extracellular and intracellular media; and iii) micelles of sodium dodecyl sulfate (SDS) to mimic the lipid bilayer environment.[qv: 34–36] The latter is useful for steady‐state and transient absorption measurements (vide infra) that are difficult to perform in intact cells. We selected SDS as it spontaneously forms mixed micelles upon combination with single‐tailed surfactants with oppositely charged head groups.[qv: 32,37,38] In the UV–vis absorption spectra (Figure 1b), we observe a large bathochromic shift when passing from DMSO to water (22 nm), in good agreement with the 26 nm shift computed at the TDDFT level, accompanied by the vanishing of the vibronic structure. On the other hand, suspension of ZIAPIN2 in the SDS micellar dispersion shows a reduced spectral shift and a marked recovery of the vibronic structure (peaking at 444 and 422 nm), which can be assigned to a more rigid conformation adopted by the azobenzene upon interaction with the alkyl chains of the surfactant.[qv: 39] PL spectra (Figure 1c) are more sensitive to the local environment as emission occurs after re‐equilibration within the solvent cage. Accordingly, PL spectra reveal dramatic changes in both spectral position and relative emission quantum yield. Interestingly, we note the development of a new red‐shifted and structureless PL band centered at 600 nm upon addition of water to the DMSO solution (Figure 1c and Figure S1a, Supporting Information), affording progressively higher relative intensity than in DMSO (up to almost one order of magnitude). Such a behavior, alongside the progressive and marked increase of the Stokes shift (100 nm; Figure S1b, Supporting Information) cannot be explained either by solvatochromic effects[qv: 40–42] or by TDDFT calculations performed on the single molecule level, and possibly indicates the occurrence of an excited state process. Interestingly, a propensity for ZIAPIN2 to aggregate in an anti‐parallel stacking dimer configuration is computed by using semi empirical tight‐binding quantum‐chemical calculations (i.e., geometry, frequency, noncovalent, extended TB method;[qv: 43] see Supporting Information), carried out on ZIAPIN2 dimer in trans conformation. Semi‐empirical excited state vertical transition energies (e.g., zerner's intermediate neglect of differential overlap level) were computed on the optimized dimer, resulting in an in‐ (i.e., high energy) and out‐of‐phase (i.e., low energy) splitting of the monomer bright state (see Figure S2, Supporting Information). Calculations for the Kasha‐like dimer indicate the presence of a red‐shifted and radiative active state that could be responsible for the enhanced emission at 600 nm. On the other hand, such a broad, structureless, and highly Stokes shifted PL band might also originate from an excimer.[qv: 44,45] The propensity to aggregate is further confirmed by scanning electron microscopy (SEM) images taken on azobenzene solid films drop‐cast from water solution (Figure S3a,b, Supporting Information), which show the presence of circular micelle‐like micron‐sized clusters. Conversely, when SDS is added to the aqueous buffer, ZIAPIN2 emission peaks at 570 nm, and features a threefold decrease in the relative emission intensity if compared to the water solution, suggesting that it could reacquire its non‐radiative pathway. As a reference, we acquired absorption and PL spectra in the culture medium (Dulbecco's modified Eagle's medium, see Supporting Information for cell culturing), which also display a clear red‐shift of absorption/emission of ZIAPIN2 with respect to DMSO and SDS buffer (Figure S4, Supporting Information), suggesting that ZIAPIN2 aggregation is also occurring in such aqueous medium. Time‐lapse confocal imaging on living HEK293 cells loaded with ZIAPIN2 in DMSO (Figure 1d) confirms the affinity of the molecule for lipid bilayers and its dynamics of incorporation. The sequence of bright‐field and fluorescence images (488 nm excitation, 500–700 nm emission) demonstrates an increase in signal intensity at cell membrane level lasting approximately 7 min, and subsequently reaching a stable plateau (see Figure S5a,b, Supporting Information, for the 3D z‐stack images of ZIAPIN2 fluorescence in HEK293 cells and co‐localization data, and Figure S5c, Supporting Information, for the fluorescence intensity evolution during time‐lapse acquisitions).

Steady‐state absorption measurements of ZIAPIN2 in solution under an actinic light allow the assessment of the ensemble dynamics of isomerization. In **Figure** **2**a,c,e, we present the absorption spectra of ZIAPIN2 under illumination with a blue LED (470 nm), alongside the photoswitching dynamics in DMSO, SDS 100 mm, and water (Figure 2b,d,f). In DMSO (Figure 2a,b), we observe a trans→cis isomerization reaction that leads to a cis‐rich photostationary state in 200 ms (extracted time constant τ = 60 ms) and a thermal cis→trans back relaxation that is completed in ≈20 s (τ = 3 s). The presence of the cis species is confirmed by the rising of the two absorption bands at 370 and 550 nm (Figure 2a), in good agreement with TDDFT calculations. Standard rate‐equations allow extracting an isomerization rate *K* = 4 × 10^3^ cm^2^ J^−1^. This is indeed a measure of the trans‐isomer absorption cross‐section normalized by photon energy, yielding a value of 2 × 10^−15^ cm^2^. This value is consistent with the direct evaluation by standard transmission measurements, validating the isomerization experiments. The thermal rate of back conversion from cis to trans is 0.06 s^−1^. The absorption band centered at 500 nm accounts for the residual trans population (30%). In water (Figure 2e,f), ZIAPIN2 photoisomerization is totally suppressed, likely owing to the formation of micellar aggregates that hinder molecular switching. In SDS (Figure 2c,d), however, we see a recovery of the fast photoswitching ability (τ = 60 ms), although the photostationary state contains only 10% of the cis isomer (Figure 2a and inset). The reason of such decreased isomerization yield in SDS compared to DMSO can be ascribed to the convolution of two effects, namely: i) ZIAPIN2 molecules are not fully incorporated by SDS, with a fraction remaining in water and forming non‐isomerizing aggregates; ii) a more constrained conformational freedom experienced by ZIAPIN2 inside the micellar environment than when solubilized in DMSO.[qv: 32] The time‐dependent quenching of trans isomer PL upon illumination is an alternative proof of the isomerization reaction, based on the notion that isomerization competes with radiative recombination and the cis isomer is poorly or non‐emitting.[qv: 34] This opens up the possibility to follow photoisomerization in biological media where absorption measurements, due to high scattering and reduced optical density, would not be feasible. Therefore, we recorded the isomerization reaction also in HEK293 living cells treated with 25 µm ZIAPIN2 by using a micro‐PL set‐up. The PL images (Figure S6a,b, Supporting Information) indicate that the photoinduced PL quenching occurs almost totally in the membrane region, while residual ZIAPIN2 molecules present in the aqueous extracellular buffer do not seem to undergo isomerization. This confirms that lipid environment allows restoration of ZIAPIN2 photoswitching ability.

**Figure 2:**
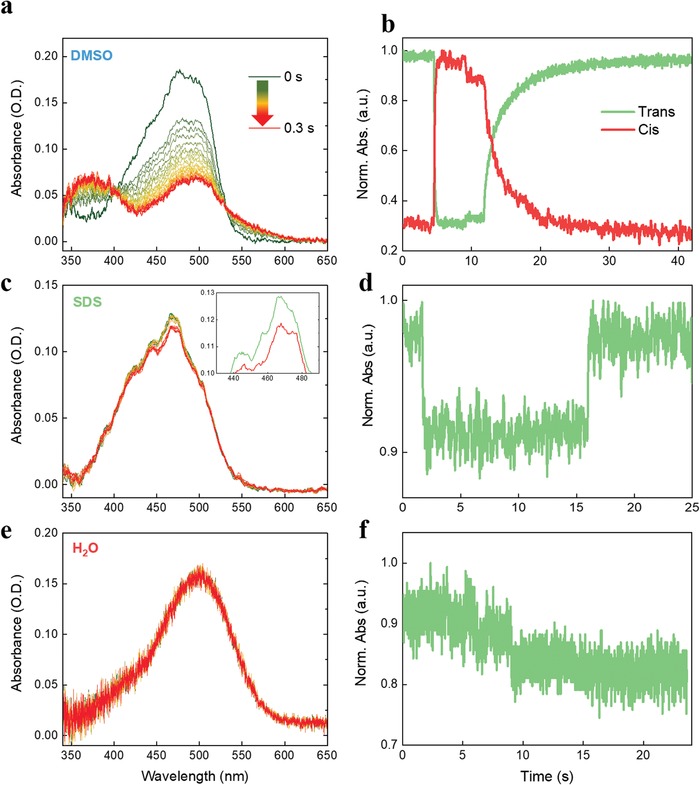
UV–vis absorption spectra of ZIAPIN2 in a) DMSO, c) SDS (100 mm), and e) water taken under illumination with a blue LED (470 nm). The inset in Figure 2c highlights the 10% decay in the trans absorption of ZIAPIN2 in SDS. Isomerization kinetics of ZIAPIN2 in DMSO taken at b) 490 nm, d) SDS (100 mm), and f) water as a function of illumination time.

In electrophysiology experiments, neurons reacted to short burst of light, between 20 and 200 ms. The prompt response seems in contradiction with the time evolution measured in solution, taking place in seconds. One should however notice that steady‐state measurements allow accessing the isomer fraction population in the ensemble. In order to gain insights into the molecular isomerization event (expected to occur in the picosecond time scale[qv: 12]) it is necessary to employ ultrafast techniques.[qv: 46] We carry out a transient absorption (TA) experiment where a pump pulse brings population to the excited state, while a broadband probe pulse interrogates this state and tracks its temporal evolution. With this technique, we studied the ZIAPIN2 photodynamics in the three media of choice, namely water, DMSO, and SDS. The TA spectra at early delay‐times (i.e., pump‐probe delay 1 ps, **Figure** **3**a) in all three media shows three main features: i) a positive peak centered at 470–500 nm that can be attributed to ground state bleaching (S_0_→S*, PB); ii) another positive peak at 570–610 nm that can be linked to stimulated emission (S*→S_0_, SE), and iii) a negative feature at around 700 nm due to photoinduced absorption from excited states (S*→S_n_, PA). To obtain quantitative insights into the complex convolution of the trans and cis spectra, we employed a global analysis algorithm. We describe our experimental data with two components (Figure 3b,c), with different decay associated spectra (DAS) and kinetics. In DMSO and SDS, the photodynamics is well consistent with the isomerization reaction,[qv: 12] in which the first component is the trans isomer that decays (τ = 6 and 12 ps for DMSO and SDS, respectively) into the second component, the long‐living cis isomer metastable state (3 and 6 ns for DMSO and SDS, respectively). Conversely, in water, the first component evolves rapidly (τ = 2 ps) into a second red‐shifted component (about 30 nm) with a relatively short lifetime (τ = 20 ps). This latter scenario can be consistent with the photoinduced formation of excimers[qv: 44] within the micellar aggregates. The first component can be related to the trans isomer that decays in the excimeric red‐shifted state (second component) via vibrational relaxation. The second component is therefore assigned to the excimer state. This photophysics landscape (see Figure 3f for a sketch) is corroborated by the experimental transient dynamics at probe wavelengths corresponding to ground state bleaching (PB at 500–520 nm, Figure 3d) and at 550–580 nm (Figure 3e). The former shows an almost complete extinction of the PB signal in water due to rapid ground state re‐population, while the latter highlights both the long‐living nature of the photogenerated cis species in DMSO and SDS and the fast (20 ps) deactivation of the excimeric state in water. By working out the intrinsic radiative lifetime of 1.5 ns using the Strickler–Berg relation[qv: 47] and assuming isomerization as the only non‐radiative decay pathway, we can estimate that the PL quantum yield (PLQY) in DMSO and SDS should not exceed 0.4% and 0.8%, respectively. This is in good agreement with the experimental data, as reported in Figure 1c, suggesting a ratio PLQY_DMSO_/PLQY_SDS_ = 0.43. On the other hand, the significant deviation between the expected ratio PLQY_DMSO_/PLQY_H2O_ (0.3) and the experimental value (0.12) for ZIAPIN2 in water (i.e., the enhanced PLQY in water) confirms the kicking in of a different deactivation mechanism in the aqueous buffer. We assign the breakdown of the Strickler–Berg rule to the formation of an aggregate excited state, possibly an excimer, with higher radiative rate. The findings in SDS are consistent with our steady‐state spectroscopic data and suggest that ZIAPIN2 performs ultrafast isomerization reaction in membrane. The tiny changes observed in the ensemble measurements in SDS/cells are likely due to the presence of a mixed population in which only a fraction of ZIAPIN2 molecules is protected by water.

**Figure 3:**
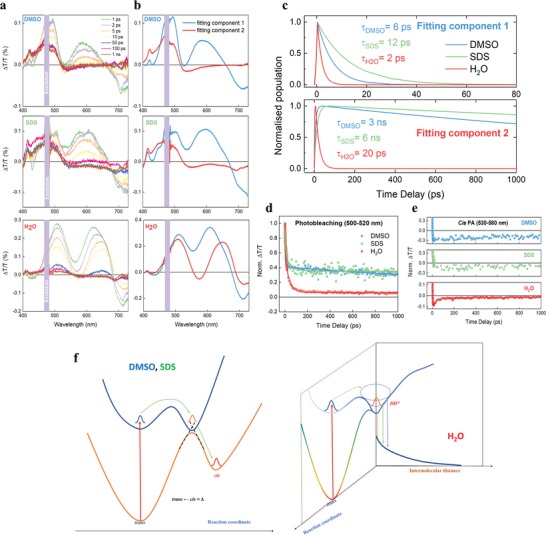
a) TA spectra in DMSO (top panel), SDS (100 mm, central panel) and water (bottom panel). The system was excited at 470 nm (π–π*) and probed with a white light super continuum. The three solutions (25 µm) were flowed by means of a peristaltic pump, to permit relaxation from the cis state. b) Global analysis of the experimental TA spectra into two fitting components. The first and second components observed in DMSO and SDS can be associated with the trans and cis isomer spectra, respectively. In H_2_O, this latter component can be linked to the excimer formation. c) Population evolution (normalized) of the two SVD components in DMSO, SDS, and H_2_O. d) TA experimental dynamics of ZIAPIN2 in DMSO, SDS 100 mm, and H_2_O (25 µm) excited at 470 nm and probed in the photobleaching region (500–520 nm) and e) in the 530–580 nm region. Solid lines represent the best‐fit of the experimental data. f) Sketch of the photodynamics in DMSO, SDS, and water as suggested by the TA data.

To further investigate the biological effect previously demonstrated in neurons both in vitro and in vivo,[qv: 34] here we study the photoreaction in a model system, namely non‐excitable HEK293 cells. These have an intrinsically low concentration of ion channels that enables to probe the opto‐mechanical effect on the cell membrane free from other effects possibly present in neurons. We proceed by loading with a DMSO/water solution of ZIAPIN2 (25 µm) in HEK293 cells, which represent a well‐established cellular model. Possible cytotoxicity of ZIAPIN2 was evaluated by using the Alamar Blue standard test, which indicates that exposure to ZIAPIN2 does not have an appreciable impact on cell proliferation (Figure S7, Supporting Information). We measured the light‐induced change in the resting plasma membrane potential (*V*_m_) by applying the patch‐clamp technique in current clamp mode (*I* = 0) (Figure S8, Supporting Information). In agreement with what has been found in neuronal cells,[qv: 34] photostimulation of ZIAPIN2‐loaded HEK293 cells with either short (20 ms) or long (200 ms) visible light pulses (470 nm, 50 mW mm^−2^) also generated a significant hyperpolarization of *V*_m_ that appeared immediately at the light onset and slowly returned to the physiological resting values after a slight rebound depolarization phase after the light offset (Figure S8a,b, Supporting Information). In our previous study, we have assigned this potential modulation to a photoinduced thinning/thickening of the cell membrane leading to an increase/decrease of the membrane capacitance.[qv: 34] To link ultimately such an effect to the ZIAPIN2 ultrafast photoreaction occurring in the membrane environment, we therefore recorded the |Δ*V*_m_| of hyperpolarization and depolarization as a function of the illumination wavelength for 20 and 200 ms light stimulation (**Figure** **4**c–f). Remarkably, we observed an almost perfect overlap of |Δ*V*_m_| with the absorption spectrum of ZIAPIN2 in SDS micelles, further confirming that the light‐induced modulation of *V*_m_ is indeed related to the trans→cis photoreaction enabled by ZIAPIN2 internalization in the plasma membrane.

**Figure 4:**
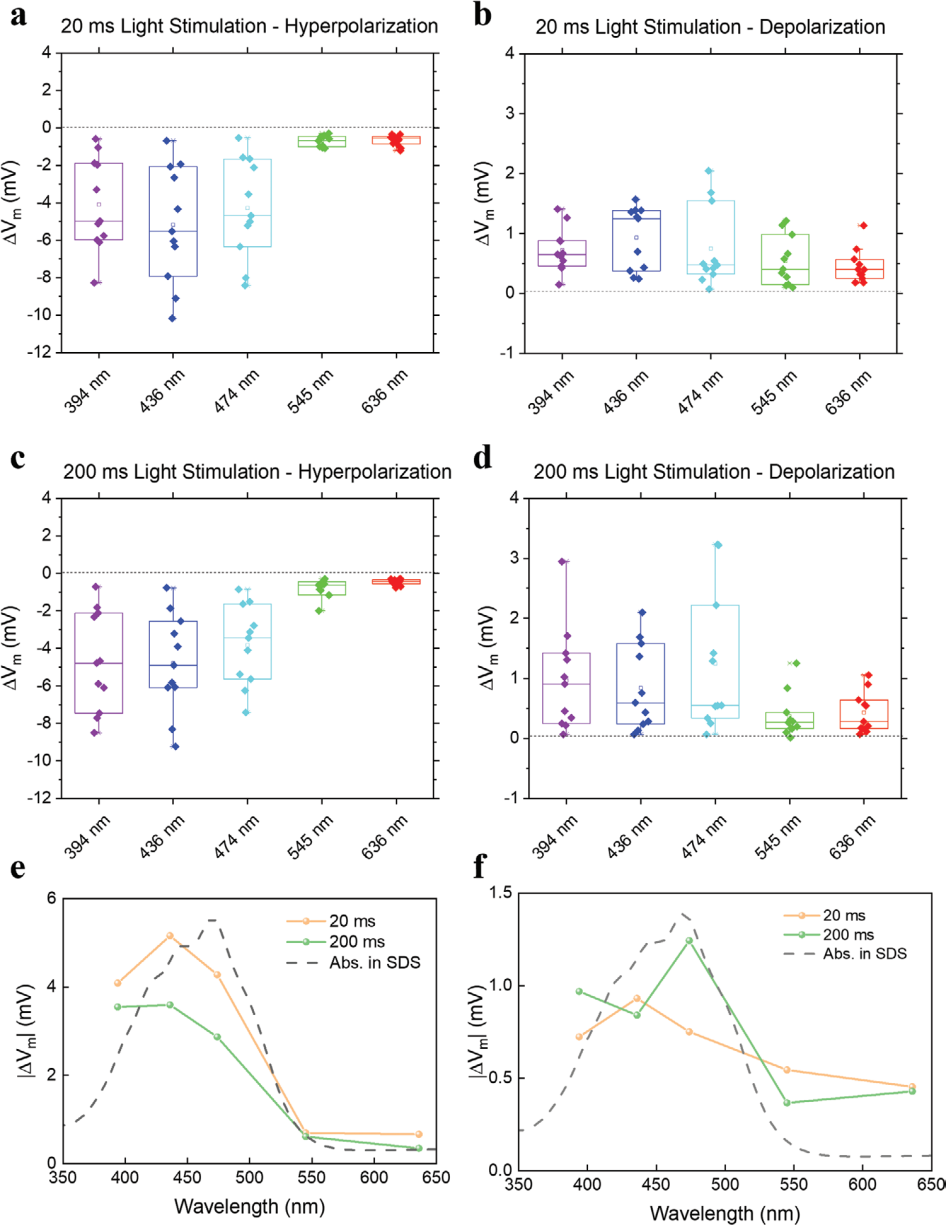
a–d) Box plots of the parameters investigated for voltage membrane modulation analysis as a function of illumination wavelength. Hyperpolarization (a,c) and depolarization (b,d) changes of HEK293 cells or exposed to DMSO/ZIAPIN2, subjected to 20 or 200 ms light stimulation (*n* = 14, 18, 20 for CTRL, DMSO, and ZIAPIN2, respectively). Hyperpolarization and depolarization were measured as the minimum and maximum voltage, respectively, reached within 350 ms from the light‐onset. e,f) Action spectrum versus wavelength of the |Δ*V*| of hyperpolarization (e) and depolarization (f) in HEK293 treated with 25 µm ZIAPIN2, for both short (20 ms) and long (200 ms) light stimuli. We also report the absorption spectrum of ZIAPIN2 in SDS for comparison (dotted grey line). All experiments were carried out at 24 ± 1 °C. **p < 0.01; ***p < 0.001; ****p < 0.0001, Kruskal–Wallis test.

To summarize, the observed aggregation of photochromic molecules in water, where they lose their ability to photoisomerize, might represent an impediment to their application in biology as water is the main component of biological environments. However, here we find that it is not true if the molecules have a preferential affinity for the cell membrane. In particular, we show that an amphiphilic azobenzene (ZIAPIN2) with non‐covalent affinity for cell membranes can act as an efficient, fast, and reversible modulator of the membrane potential via visible light. By applying steady‐state and time‐resolved ultrafast spectroscopies, we note that in water a strong aggregation occurs, effectively hampering photoisomerization and opening up a deactivation path likely mediated by excimer formation. Alternatively, internalization in the cell membrane prevents self‐aggregation and restores the intrinsic ultrafast response of the photo switch. This, ultimately allows light‐triggering a large hyperpolarization in HEK293 cells whose magnitude follows the absorption profile of the trans state in membrane and, remarkably, permits evoking biological photoinduced effects in vivo.[qv: 34] Such actuators are new tools that could contribute to the development of opto‐neuroscience, biophotonics, and photopharmacology.

## Conflict of Interest

The authors declare no conflict of interest.

## Supporting information

Supporting information
