# Supplementary material for: Membrane Environment Enables Ultrafast Isomerization of Amphiphilic Azobenzene
Source: Adv Sci (Weinh). 2020 Mar 6;7(8):1903241. doi: 10.1002/advs.201903241 (PMC7175258; doi:10.1002/advs.201903241)
Supplement: Supplementary file 1 — Supporting information [file ADVS-7-1903241-s001.pdf]

Copyright WILEY-VCH Verlag GmbH & Co. KGaA, 69469 Weinheim, Germany, 2020.

ADVANCED SCIENCE
Open Access

Supporting Information

for *Adv. Sci.,* DOI: 10.1002/advs.201903241

Membrane Environment Enables Ultrafast Isomerization of Amphiphilic Azobenzene

Giuseppe Maria Paternò, Elisabetta Colombo, Vito Vurro, Francesco Lodola, Simone Cimò, Valentina Sesti, Egle Molotokaite, Mattia Bramini, Lucia Ganzer, Daniele Fazzi, Cosimo D'Andrea, Fabio Benfenati, Chiara Bertarelli, and Guglielmo Lanzani**

Supplementary Information for

Membrane Environment Enables Ultrafast Isomerisation of Amphiphilic Azobenzene

G. M. Paternò*[1], E. Colombo*[2,3], V. Vurro[1,4], F. Lodola[1], S. Cimò[5], V. Sesti[5], E. Molotokaite[1], M. Bramini[2,3†], L. Ganzer[4], D. Fazzi[6], C. D'Andrea[1,4], F. Benfenati[2,3], C. Bertarelli°[5], G. Lanzani°[1,4]

[1]Center for Nano Science and Technology, Istituto Italiano di Tecnologia, Via Pascoli 70/3, 20133, Milano, Italy

[2]Center for Synaptic Neuroscience and Technology, Istituto Italiano di Tecnologia, Largo Rosanna Benzi 10, 16132 Genova, Italy

[3]IRCCS Ospedale Policlinico San Martino, Largo Rosanna Benzi 10, 16132 Genova, Italy

[4]Dipartimento di Fisica, Politecnico di Milano, Piazza L. da Vinci 32, 20133 Milano, Italy

[5]Dipartimento di Chimica, Materiali e Ingegneria Chimica "Giulio Natta", Politecnico di Milano, Piazza L. da Vinci 32, 20133 Milano, Italy

[6]Department of Chemistry, Institut für Physikalische Chemie, University of Cologne, Luxemburger Str. 116, D -50939 Köln, Germany

*equally contributing authors

[†]Present address: Department of Applied Physics, Faculty of Sciences, University of Granada, C/Fuentenueva s/n, 18071-Granada, Spain

°Corresponding authors: Guglielmo Lanzani, email: guglielmo.lanzani@iit.it ; Chiara Bertarelli, email: chiara.bertarelli@polimi.it

Experimental section

Synthesis of ZIAPIN2. Unless otherwise stated, all chemicals and solvent were commercially available and used without further purification. Thin layer chromatography (TLC) was performed using silica gel on aluminum foil (Sigma Aldrich). ^{1}H and ^{13}C NMR spectra were collected with a Bruker ARX400. Mass spectroscopy was carried out with a Bruker Esquire 3000 plus.

4-[2-(4-aminophenyl)diazen-1-yl]aniline (1)

A mixture of Disperse Orange 3 (Sigma Aldrich, 1.21 g, 5.0 mmol) and $Na_2S\cdot 9H_2O$ (3.60 g 15.0 mmol) dissolved in 100 mL of MeOH is refluxed overnight under stirring. Then the mixture is cooled to room

temperature and the solvent is removed under reduced pressure. The resulting red powder is washed with DCM and Et_2O, the combined organic layers are collected, and the solvent is evaporated under reduced pressure, to give 540 mg of the desired product **1** as an orange powder in 51% yield.

^{1}H NMR: (400MHz, DMSO) δ 7.56 (d, J= 8.82Hz, 2H), 6.62 (d, J=8.82Hz, 2H), 5.70 (s, 4H)

4-((4-(azepan-1-yl) phenyl)diazen-1-yl)-N,N-bis(6-bromohexyl)aniline (Azo-Br2)

537 mg of **1** (2.53 mmol) is stirred in 10 ml of previously degassed acetonitrile. 2.20 g of K_2CO_3 (15.9 mmol) and 1.6 ml of 1,6-dibromohexane (10.4 mmol) are added dropwise to the reaction mixture and refluxed for 72 hours, while monitored by TLC. The reaction mixture is filtered and the solid is washed three times with diethyl ether, ethylacetate and dichloromethane. The excess of dibromohexane is removed under reduced pressure (3 10^{-1} mbar) at 70 °C. The raw material is purified by flash chromatography with silica gel using hexane: Et_2O 3:1 as eluent to give 33 mg of **Azo-Br2** (2.1 % yield) are also recovered.

1H-NMR: (400MHz, DMSO) δ (ppm) 7.63 (d, J= 8.80Hz, 4H), 6.77 (d, J= 8.80 Hz, 2H), 6.72 (d, J= 8.80Hz, 2H), 3.53 (t, -N-CH_2-, 8H), 3.35 (t, -CH_2-Br), 1.82-1.35 (m, -CH_2-, 24H); MS : 621 $(M + H)^+$

1-{6-[(4-{2-[4-(azepan-1-yl) phenyl]diazen-1-yl}phenyl)[6-(pyridin-1-ium-1-yl) hexyl] amino] hexyl} pyridin-1-ium dibromide (ZIAPIN 2)

12 mg of **Azo-Br2** are dissolved in 3 ml of pyridine and stirred at room temperature for 42 hrs. Then 3 ml of methanol are added and further stirred for 60 hrs. The excess of pyridine and methanol are removed from the reaction mixture under reduced pressure to give a solid in quantitative yield that is further washed with small portions of hexane.

^{1}H NMR: (400MHz, DMSO) δ 9.09 (d, Pyr, 4H), 8.61 (t, Pyr, 2H), 8.16 (t, Pyr, 4H), 7.62 (d, J= 8.8 Hz, Ph, 4H), 6.78 (d, J=8.8 Hz, Ph, 2H), 6.70 (d, J= 8.8 Hz, Ph, 2H), 4.60 (t, -CH_2-Pyr-, 4H), 3.54 (t, N-CH_2-, 4H), 2.97-1.24 (m, 24H). ^{13}C NMR: (400 MHz, DMSO) δ 150.29, 149.45, 145.97, 145.21, 143.00, 128.56, 124.38, 124.24, 111.65, 111.36, 61.22, 50.54, 49.50, 31.15, 27.27, 27.15, 26.75, 26.27, 25.86. MS: 618 $(M - 2Br)^+$

The spectrum of the isolated compound absorbs at nearly 470 nm. To proceed with the spectroscopic investigations and electrophysiology measurements, ZIAPIN2 was dissolved in DMSO at a concentration of 2 mM.

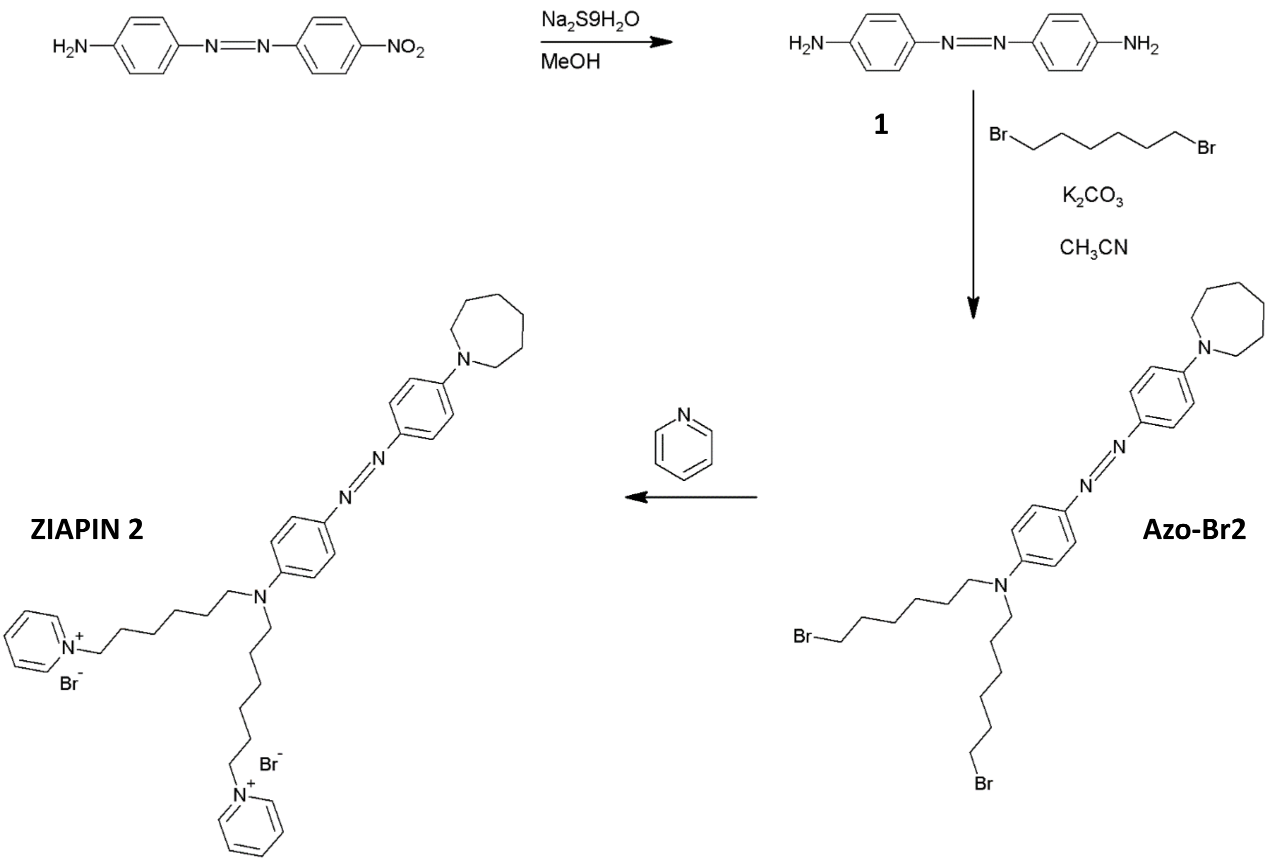

Scheme S1. Synthesis of ZIAPIN2

Quantum chemical calculations. The molecular structures of trans and cis isomers were optimized at the DFT level using the B3LYP hybrid functional combined with the Dunning's correlation consistent triple split basis set cc-pVTZ. Vibrational frequency analyses do not reveal negative eigenvalues, thus indicating that the structures are at the equilibrium configurations. TDDFT calculations were performed at the same level of theory considering up to 80 excited states each isomer. Water effects were simulated for the trans species via the continuum polarizable conductor model (CPCM); the molecule was re-optimized at the DFT/CPCM level and TDDFT/CPCM calculations were computed. All calculations were performed with Gaussian16.[1] Dimers were optimized at the semiempirical tight-binding quantum chemical method GFN1-xTB,[2] and the excited states computed at the ZINDO/S level.

Steady-state UV-Vis and PL measurements. The solutions were prepared by suspending the proper amount of ZIAPIN2 in DMSO, SDS above its critical micellar concentration (100 mM) and H_2O to obtain a concentration of 25 μM. For the UV-Vis absorption measurements, we employed both a halogen a deuterium

lamp as transmission probe. The transmitted light was focused to a fibre-fed spectrometer (Ocean Optics Maya Pro 2000). Isomerisation was triggered by means of a LED lamp centred at 470 nm at a power of 100 mW, which was placed on top of a quartz cuvette (1 mm path) containing the ZIAPIN2 solutions. The sample was illuminated uniformly. The micro-PL on HEK293 cells was acquired by exciting with a CW diode laser (excitation energy of 10 mW mm^{-2}). The emission was collected with a 50x objective (Zeiss), filtered to remove the excitation line and sent to the camera (Hamamatsu, acquisition time 100 ms).

Scanning electron microscopy. We used Tescan MIRA3 microscope. The measurements were performed at a voltage of 5 kV and backscattered electrons were detected. ZIAPIN2 solutions were drop-cast on top of silicon substrates and let to dry. The sample were covered with carbon paste to improve conductivity.

Confocal microscopy. Live HEK cells were loaded with CellMask^{TM} Deep Red plasma membrane stain (1 µl/ml; Thermo Fischer) for the evaluation of the localization of ZIAPIN2 at the plasma membrane. Cells were cultured as described in the electrophysiology section (see below). Briefly, HEK cells were exposed to ZIAPIN2 in DMSO (25 µM) for 7 min; the molecule was then washed away, and cells were treated with CellMask^{TM} Deep Red, following the manufacturer instructions. Coverslips with cells were washed with fresh medium and mounted on a confocal laser scanning microscope (CLSM) Leica SP8 (Leica Microsystem) for live-cell z-series stack acquisition of consecutive confocal sections (40x 1.4 NA objective). For time lapse experiments, HEK cells were loaded with 25 µM ZIAPIN2 in DMSO and imaged in 2D-confocal mode every 30 seconds for 10 min; bright-field images were acquired to maintain the focus and visualize the localization of ZIAPIN2 overtime.

Ultrafast time-resolved spectroscopy. For the femtosecond TA measurements, we used a Ti:Sapphire laser with a repetition rate of 1 kHz and a pulse width 100-150 fs. We excited the solutions with a wavelength of 470 nm that was generated by means of an optical parameter amplifier and probed with a white light beam generated by a CaF_2 plate. The excitation energy was of ≈ 20 nJ and the beam spot size of ≈ 200 µm in diameter. We had to circulate the solution containing the sample because ZIAPIN2 recovery time to the *trans* ground state far exceeds the time interval between pulses (set by the laser repetition rate). Circulation assures that a “fresh” non-excited molecule is measured by each pulse. The solutions were circulated by means of a peristaltic pump (200 rpm).

Electrophysiology. Standard patch clamp recordings were performed using Axopatch 200B (Axon Instruments) coupled to an inverted microscope (Nikon Eclipse Ti). HEK293 cells (purchased from ATCC®) were cultured in Dulbecco's modified Eagle's medium (DMEM) supplemented with 10% Fetal Bovine Serum (FBS), 100 U/ml Penicillin and 100 µg/ml Streptomycin and maintained in a humidified incubator at 37°C with 5% CO_2. HEK293 cells (laboratory passage 20–22) seeded on bare glass were measured in whole-cell configuration with freshly pulled glass pipettes (3–6 MΩ), filled with the following intracellular solution [mM]: 12 KCl, 125 K-Gluconate, 1$MgCl_2$, 0.1 $CaCl_2$, 10 EGTA, 10 HEPES, 10 ATP-Na_2. The extracellular solution contained [mM]: 135 NaCl, 5.4 KCl, 5 HEPES, 10 Glucose, 1.8 $CaCl_2$, 1 $MgCl_2$. Only single HEK293 cells were selected for recordings. Acquisition was performed with pClamp-10 software (Axon Instruments). Membrane currents were low pass filtered at 2 kHz and digitized with a sampling rate of 10 kHz (Digidata 1440 A, Molecular Devices). Data were analyzed with Clampfit (Axon Instruments) and Origin 8.0 (OriginLab Corporation). HEK293 cells were incubated for 8 minutes at 37 °C with the azobenzene molecule and washed with the same physiological solution used for Patch-Clamp recordings. The light source for excitation was provided by a LED system (Lumencor Spectra X) fibre-coupled to the fluorescence port of the microscope; the illuminated spot on the sample has an area of 0.23 mm^2. Cyan emitting LED was used as light source, characterized by maximum emission wavelength at 470 nm and at a photoexcitation density of 50 mW/mm^2, as measured at the output of the microscope objective (Pobj). We also irradiated at different wavelengths to record action spectra. For this, we employed a Lumencor Spectra light engine® generation-III (8 LED light sources). Data are represented as mean ± MSE. All experiments were carried out at 24 ± 1 °C. $^{**}p<0.01$; $^{***}p<0.001$; $^{****}p<0.0001$, Kruskal-Wallis test.

Cytotoxicity. The Alamar Blue® is a cytotoxicity/proliferation assay that is based in the emission properties of Resazurin.[1] In particular, Resazurin is reduced by cell respiration when added to the cell culture. The reduced form of the molecule (Resorufin) has a higher emission yield at 590 nm compared to the oxidized Resazurin, allowing to study cells proliferation by monitoring the emission intensity at 590 nm from a fixed volume of Resazurin. In such procedure, Alamar Blue is firstly added to DMEM without phenol red (Alamar Blue® volume = 10% of the whole volume of cellular buffer). A fixed volume of this solution (500 µl) is then added to the cellular buffer and let to react with cells (3 hours). Finally, three aliquots (100 µl) are taken from each samples for emission measurements (PL wavelength 590 nm). Each emission point is

acquired at 24, 48, 72 and 96 hours after ZIAPIN2 incubation. Control measurements were taken on Alamar Blue treated HEK cells without ZIAPIN2.

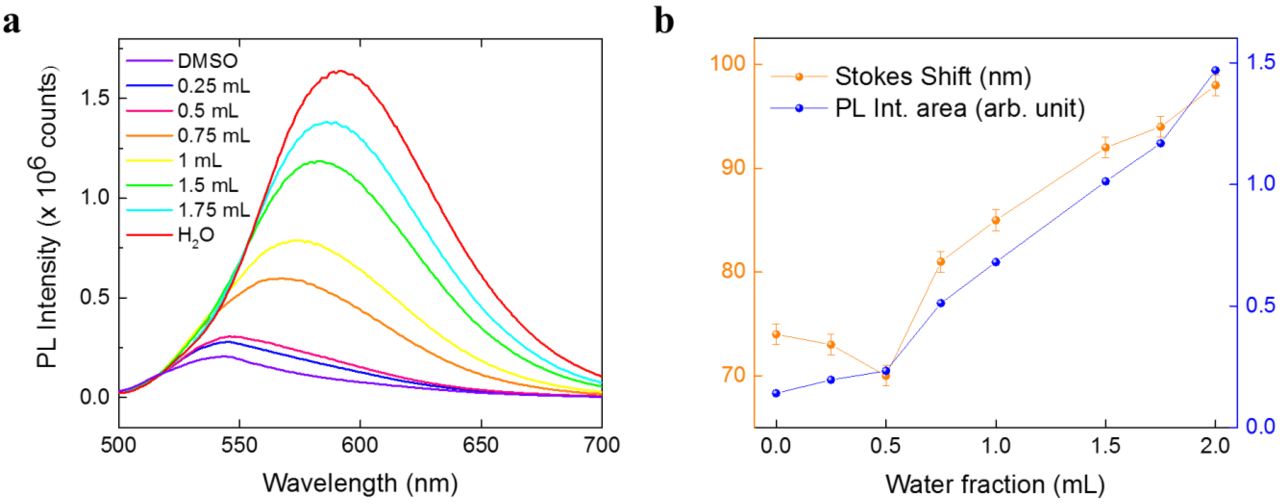

Figure S1. a, PL spectra of ZIAPIN as a function of added water volume. To establish a quantitative relationship among the relative quantum yields, sample volumes were kept constant and the obtained PL spectra were normalised to lamp intensity and ground state absorption. **b,** Stokes shift and integrated PL area (normalised to absorbance) as a function of water volume.

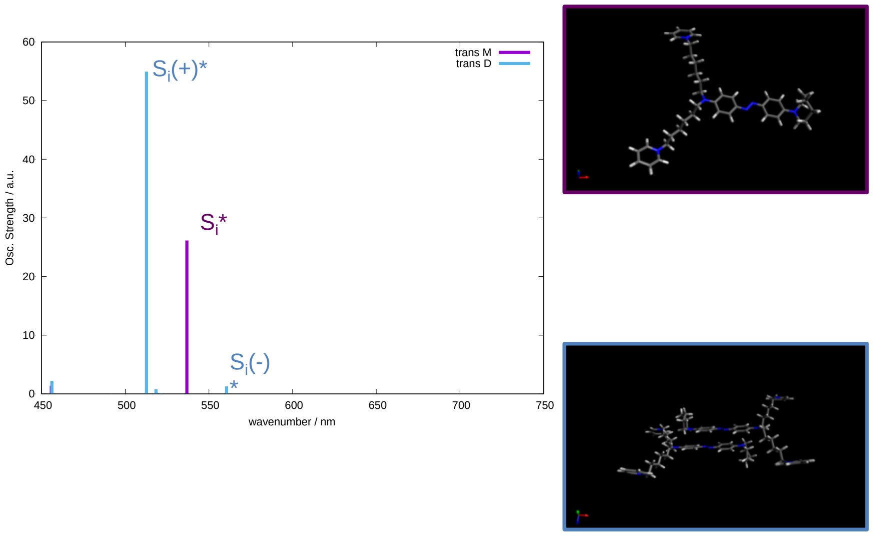

Figure S2. Computed vertical electronic transitions (ZINDO/S) for the monomer and dimer of ZIAPIN2, as optimised at the GFN1-xTB level.

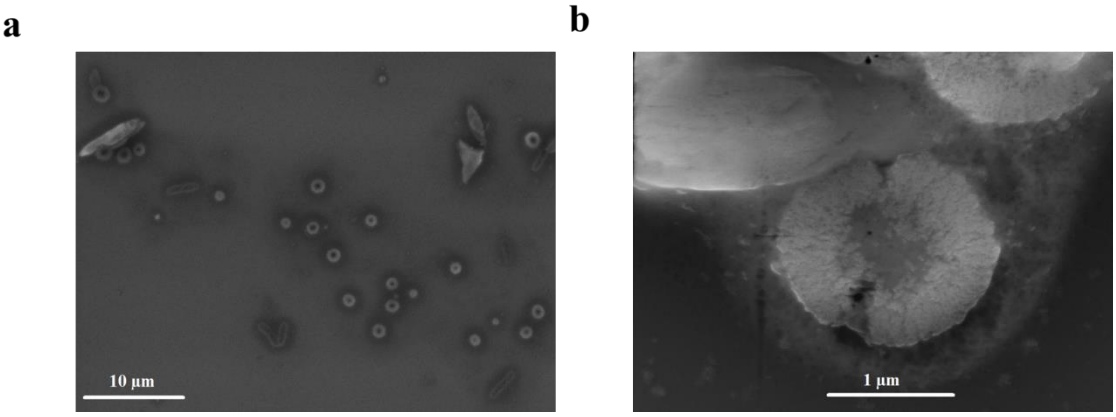

Figure S3. a,b, Scanning electron microscopy (SEM) images of ZIAPIN micellar aggregates formed upon suspension of the molecule in water. For these measurements, the ZIAPIN solution in water (25 µM) was drop-cast on a silicon substrate.

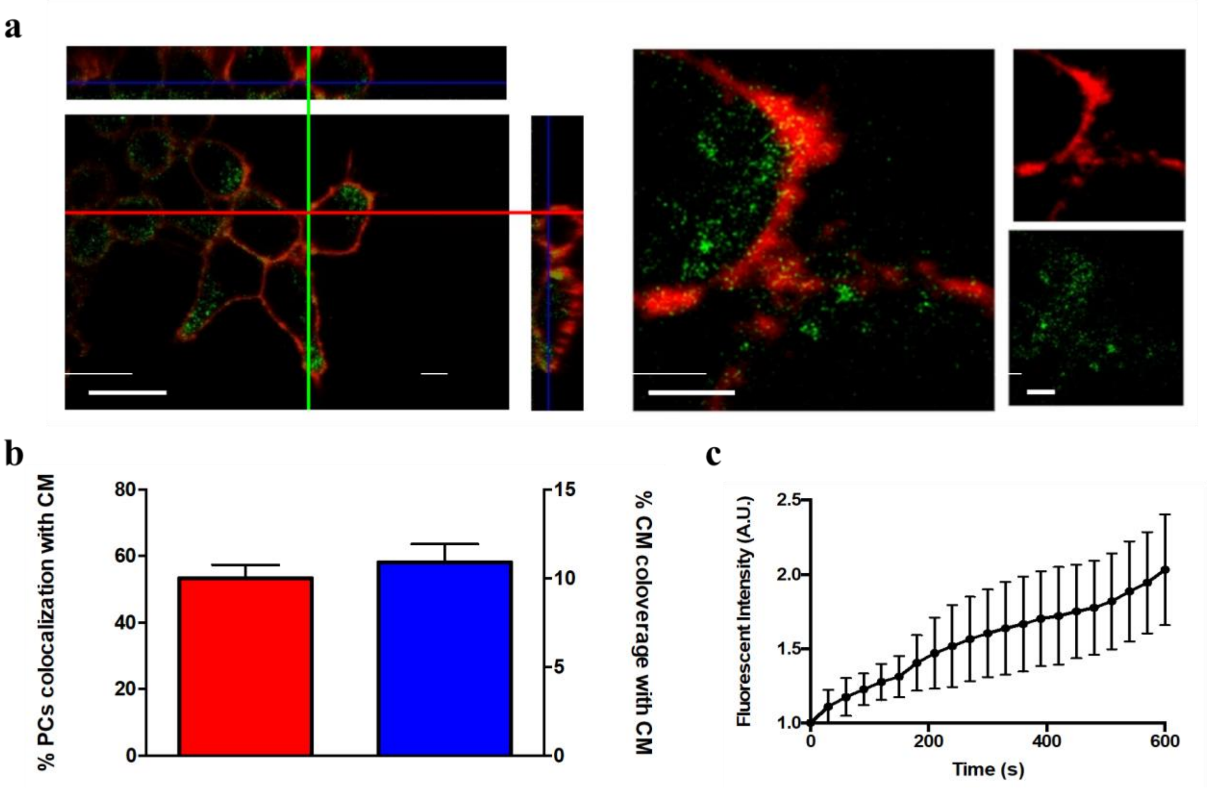

Figure S4. a, Representative confocal 3D-z-stack images of ZIAPIN2 fluorescence in HEK293 cells (green) and its co-localization with Cell Mask (CM), an amphiphilic fluorescent marker of the cell membrane (red) (left panel). Merged (left) and individual (right) images are shown. **b,** quantification of the co-localization of ZIAPIN2 with CM and its coverage. Scale bars, 10 and 20 µm for large and small panels, respectively. **c**, quantification of the fluorescence intensity during time-lapse acquisitions (see **Fig. 1d**, normalised values, 1 = starting fluorescence at time 0).

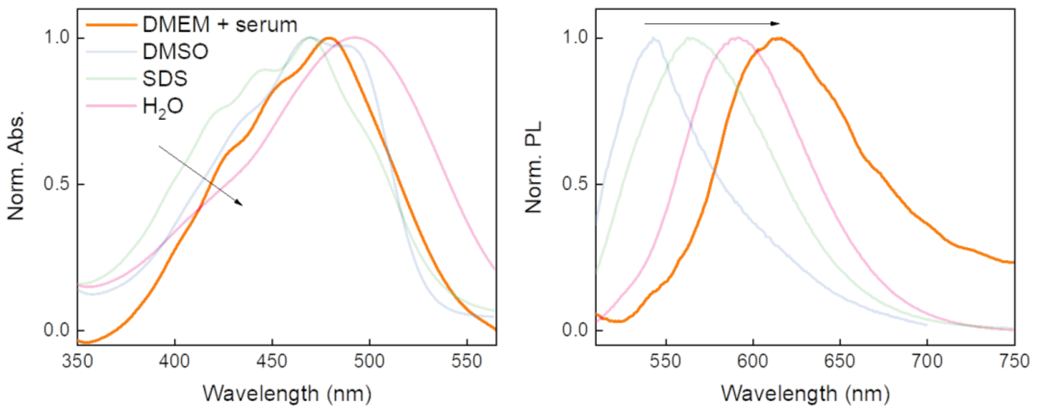

Figure S5. Absorption and emission for ZIAPIN2 in DMEM + serum. Spectra were corrected by the absorption and emission of phenol red. The enhancement of the vibronic progression and the blue-shifted absorption with the respect of pure water can be attributed to the presence of lipophilic components in the DMEM buffer (i.e. bovine serum). On the other hand, the highly red-shifted PL (620 nm) and broad PL indicate the formation of ZIAPIN2 aggregates in DMEM.

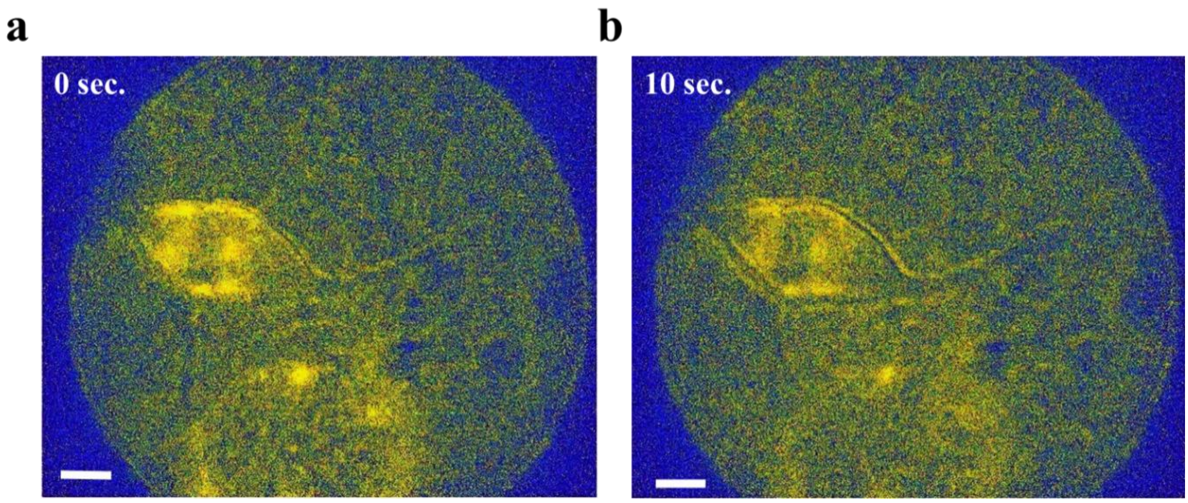

Figure S6. a,b, Micro-PL images of the HEK293 cells with ZIAPIN2 at 0 sec. at 10 sec illumination, respectively (bars 10 µm). For the measurements in cuvette, we employed a fluorimeter equipped with a blue LED (470 nm), and took the PL signal at the peak maxima (resolution 20 ms). For the PL measurements in HEK cells, we used a micro-PL set-up, equipped with an objective (40x) and a CW laser (450 nm). In this case, we had to increase the acquisition time to 100 ms to obtain a good signal-to-noise ratio.

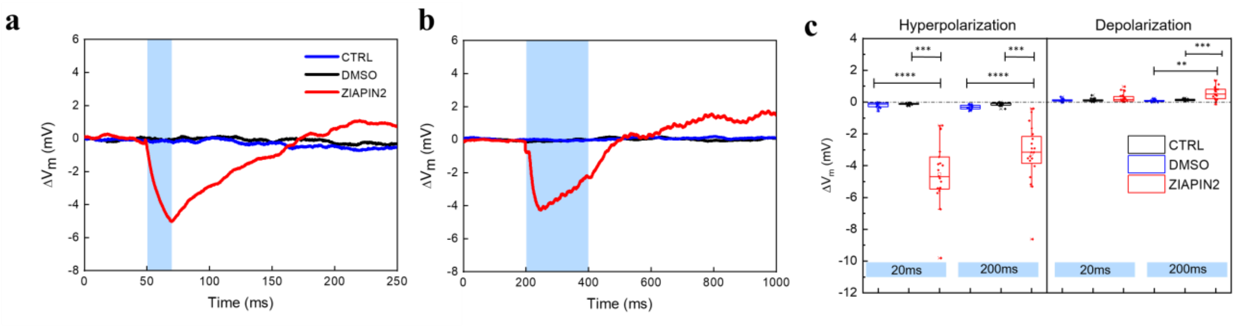

Figure S7. a,b, Representative current clamp traces showing HEK293 cells membrane potential variation in presence of 25 μM ZIAPIN2 when photostimulated with short (20ms) and long (200 ms) visible light pulses, represented by cyan shaded areas, at the light power density of 50 mW/mm2. Traces recorded on untreated (CTRL) and vehicle-treated (DMSO) cells are also shown. c, Box plots of the parameters investigated for voltage membrane modulation analysis. Hyperpolarization (left) and depolarization (right) changes of either untreated (CTRL) HEK293 cells or exposed to DMSO/ZIAPIN2, subjected to 20 or 200 ms light stimulation (n° = 14, 18, 20 for CTRL, DMSO and ZIAPIN2, respectively). Hyperpolarization and depolarization were measured as the minimum and maximum voltage, respectively, reached within 350 ms from the light-onset

[1] R. Hamid, Y. Rotshteyn, L. Rabadi, R. Parikh, P. Bullock, *Toxicol. Vitr.* **2004**, *18*, 703.
